# Imaging Features of Urachal Cancer: A Case Report

**DOI:** 10.3389/fonc.2019.01274

**Published:** 2019-11-26

**Authors:** Xiaoyan Chen, Chunsong Kang, Mingxia Zhang

**Affiliations:** ^1^Department of Ultrasound, Shanxi Academy of Medical Sciences, Shanxi DAYI Hospital, Taiyuan, China; ^2^Department of Clinical Laboratory Medicine, Shanxi Academy of Medical Sciences, Shanxi DAYI Hospital, Taiyuan, China

**Keywords:** urachus, adenocarcinoma, bladder, ultrasonography, computed tomography

## Abstract

Urachal adenocarcinoma originates from the space of Retzius. It is a rare but aggressive neoplasm. Typical imaging findings of urachal cancer are difficult to find; this report provides an ultrasonographic (US) and computed tomographic (CT) description of the above. We present a case of a 45-year-old male patient presenting with painless hematuria of 1 week's duration and display the US and CT images. Imaging shows: (1) a solid, ill-defined, irregularly shaped mass invading the bladder wall located between the dome of the bladder and the abdominal wall, and which underwent significant enhancement; (2) hypo-echoic, heterogeneous echopattern with some calcifications and (3) patchy and short-line blood flow signals within the mass. Final histopathology confirmed urachal adenocarcinoma.

## Introduction

The urachus is a tubular structure extending from the dome of the bladder to the allantois in early embryonic development. After birth, it becomes a fibrous cord located in the loose connective tissue between the transverse fascia and the peritoneum (i.e., within the space of Retzius) (1–4). Failure of complete luminal obliteration has been described in up to one-third of adults and can rarely lead to various anomalies including cysts, fistulae, diverticula, or, rarely, malignant transformations (2, 5).

Based on the statistics of the last decade, the case reports of urachal cancer are still rare each year since the seminal studies by Begg in the 1930s (6). Urachal carcinoma accounts for ~0.1% of all malignant tumors and 0.35–0.7% of all bladder tumors (7). It is usually undiscovered for a long time because the early stages are often asymptomatic. It may be identified incidentally during an imaging examination performed for other reasons or when symptoms appear at advanced stages (4, 8–10). Imaging examinations, such as ultrasonography (US), computed tomography (CT), and magnetic resonance imaging (MRI) play an important role in the workup of urachal tumors (11).

## Case Presentation

A 45-year-old man presented to our hospital with 1 week of painless hematuria with no obvious cause. He was previously healthy with no known diseases. Physical examination showed no obvious abnormalities.

Urinalysis was positive for red blood cells, 814 × 10^6^/L and white blood cells, 3 oun^6^/L. Tumor marker determination showed carcinoembryonic antigen (CEA) 2.8 × 10^−3^ ng/L (normal range: 0–5 × 10^−3^ ng/L), and prostate specific antigen (PSA) 0.48 × 10^−3^ ng/L (normal range: 0–4 × 10^−3^ ng/L).

**Figure 1:**
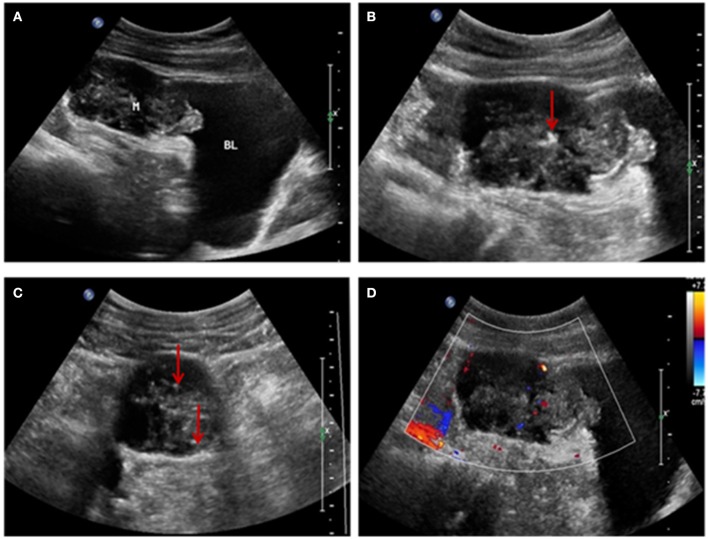
**(A)** Ultrasonographic image showing a solid mass above the bladder invading the bladder wall and with an irregular shape and uneven internal echogenicity. **(B,C)** Patchy calcification within the mass (red arrow). **(D)** Color-flow Doppler image showing patchy and short-line blood flow signals within the mass.

US examination showed a solid mass in the midline of the lower abdomen, invading the antero-inferior bladder wall. It measured 6.3 × 4.9 × 3.4 cm, with ill-defined, irregularly shaped protrusions outside the bladder extending to the umbilicus. Internal echogenicity was heterogeneous, and patchy calcifications and a small amount of point-like and short-line blood flow were observed within the tumor. No enlarged lymph nodes were noted in the abdominal cavity or retroperitoneum. We diagnosed an umbilical tumor (Figure 1).

**Figure 2:**
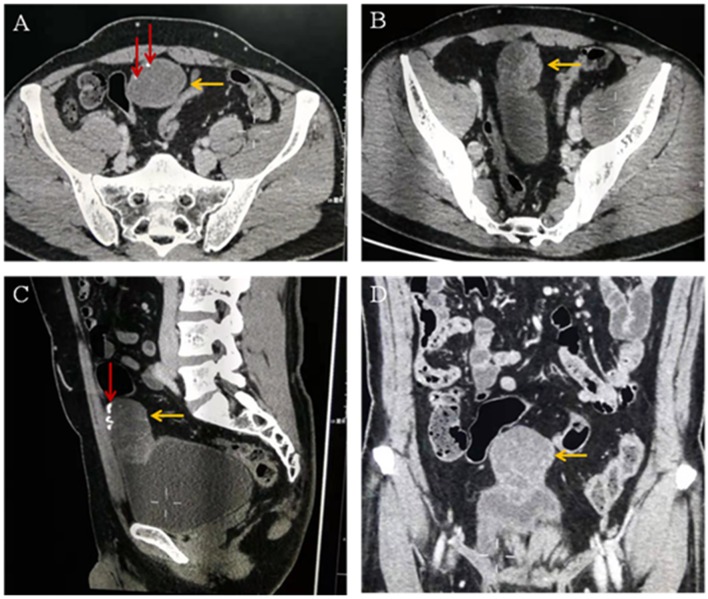
**(A–C)** Computed tomographic images showing a cystic solid mass (yellow arrow) in the anterior part of the bladder measuring 6.8 × 3.9 cm in size. The tumor invades the anterior wall and peritoneum of the adjacent bladder. Calcification (red arrow) and separation (yellow arrow) are visible within the tumor. **(D)** Enhanced computed tomographic image showing that the solid part of the mass is obviously enhanced (yellow arrow).

The diagnosis on contrast-enhanced abdominopelvic CT was also umbilical tumor. CT confirmed that there was an intra-abdominal mass with calcification, invasion of the adjacent bladder dome and peritoneum, and which underwent significant enhancement (Figure 2).

**Figure 3:**
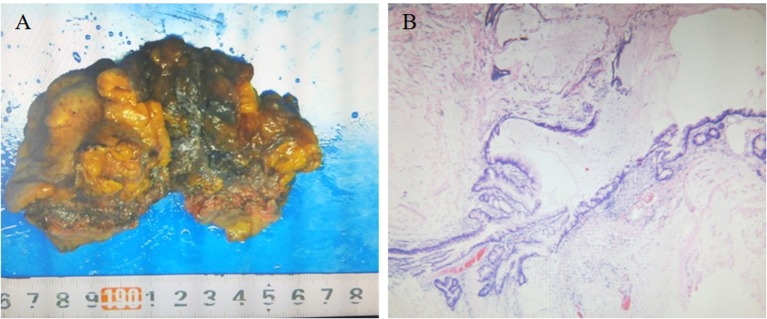
**(A)** Gross pathology showing the resected umbilical urethral tumor specimen. The swollen tumor material was brittle and adhered to the bladder wall. The cut surface was grayish white and covered with a jelly-like substance, and part of the bladder mucosa was visible. **(B)** Microscopic pathology (hematoxylin and eosin staining ×40) confirming urachal cancer (grade II mucinous adenocarcinoma) with calcification and cancer tissue infiltration into the bladder wall muscle layer.

The patient underwent surgery to remove the mass. The urachus, umbilicus, and part of the bladder dome 2.0 cm from the tumor were also resected. Intraoperative findings showed an ~5.0 × 5.5 × 5.0 cm mixed cystic and solid mass invading the dome of the bladder and adherent to the peritoneum gross pathology showed a 6.0 × 5.5 × 3.5 cm mass with a grayish-white cut surface, coated in a jelly-like substance. The tumor was friable. Histopathology of the tumor showed that part of the bladder mucosa was included in the tumor, without vascular and neurological invasion recidivism (Figure 3A). According to the Mayo staging system (3), the pathological diagnosis was urachal carcinoma (grade II mucinous adenocarcinoma) with calcification (Figure 3B). The patient received docetaxel combined with tegafur chemotherapy after surgery.

## Discussion

Urachal cancers can originate from any of the various layers of the urachus tract and arise mainly from epithelial cells; 94% of umbilical urachal cancers are adenocarcinomas, the diagnosis of our patient (6). The tumor invaded and actually eroded the bladder wall, causing painless hematuria, similar to a bladder cancer (12). Because of the non-specific clinical signs of this case, preoperative diagnosis depended on imaging.

Urachal cancer usually appears as an intra-abdominal mass adjacent to the bladder dome, extending into the space of Retzius. It can be cystic, solid, or mixed (11). US and CT can provide a general impression of the lesion, including the location, size, and invasiveness of the mass (8, 9, 13).

This report provides the characteristic US appearance of urachal cancer. The main features of this patient's ultrasonographic imaging findings were: (1) a solid mass extending between the dome of the bladder and the abdominal wall, with an irregular shape and bladder wall invasion; (2) a hypoechoic, heterogeneous echo pattern with a small amount of calcification; and (3) patchy and short-line blood flow signals within the mass. These findings are consistent with previous reports (8, 9, 13) and the key findings of this case to further enrich the imaging features of this disease.

The US and CT examinations showed that the tumor infiltration reached the bladder muscle layer, without obvious lymph node involvement or spread to other organs, consistent with intraoperative findings and surgical pathology. Urachal tumors contain mucin, and calcifications are present in 70% of cases (1, 14). The presence of calcifications in a soft-tissue mass along the course of the abdominal midline is considered pathognomonic for the diagnosis of urachal cancer (8, 9).

## Conclusion

We have described the typical imaging features of a case of urachal adenocarcinoma. Diagnosing urachal cancer is easier with imaging.

## Data Availability Statement

All datasets generated for this study are included in the article/Supplementary Material.

## Ethics Statement

The studies involving human participants were reviewed and approved by appropriate institutional of Shanxi Academy of Medical Sciences and Shanxi DAYI Hospital. The patients/participants provided their written informed consent to participate in this study. Written informed consent was obtained from the individual(s) for the publication of any potentially identifiable images or data included in this article.

## Author Contributions

XC, CK, and MZ performed image acquisition and completed the manuscript.

### Conflict of Interest

The authors declare that the research was conducted in the absence of any commercial or financial relationships that could be construed as a potential conflict of interest.
